# The Impact of Probiotics on Clinical Symptoms and Peripheral Cytokines Levels in Parkinson’s Disease: Preliminary In Vivo Data

**DOI:** 10.3390/brainsci14111147

**Published:** 2024-11-15

**Authors:** Luca Magistrelli, Elena Contaldi, Annalisa Visciglia, Giovanni Deusebio, Marco Pane, Angela Amoruso

**Affiliations:** 1 Parkinson Institute Milan, ASST G.Pini-CTO, Via Bignami 1, 20126 Milan, Italy; contaldie@yahoo.it; 2 Probiotical Research S.r.l., Via Mattei 3, 28100 Novara, Italy; a.visciglia@probiotical.com (A.V.); g.deusebio@probiotical.com (G.D.); m.pane@probiotical.com (M.P.); a.amoruso@probiotical.com (A.A.)

**Keywords:** Parkinson’s disease, neuroinflammation, probiotics

## Abstract

**Introduction.** Previous studies have shown that probiotics have positive effects on both motor and non-motor symptoms in Parkinson’s disease (PD). Additionally, in preclinical settings, probiotics have demonstrated the ability to counteract neuronal loss and alpha-synuclein aggregation, important pathological hallmarks of PD. Notably, preliminary in vitro studies have revealed the immunomodulatory properties of probiotics. This study aims to evaluate the impact of probiotics on symptoms and peripheral cytokines levels in PD patients compared to placebo. **Methods.** Patients were enrolled and blindly randomized to receive either active probiotics (comprising *Bifidobacterium animalis* subsp. *lactis* BS01 LMG P-21384, *Bifidobacterium longum* BL03 DSM 16603, *Bifidobacterium adolescentis* BA02 DSM 18351, Fructo-oligosaccharides and Maltodextrin-Group A) or placebo (Maltodextrin-Group B). Clinical evaluations and plasma levels cytokines (TNF-α, IFN-γ, IL-6, and TGF-β) were also assessed at enrollment and after 12 weeks. Anti-parkinsonian therapy remained stable throughout the study. **Results.** Forty PD patients were recruited. After 12 weeks, Group A showed significant improvement in motor symptoms (UPDRS III: 13.89 ± 4.08 vs. 12.74 ± 4.57, *p* = 0.028) and non-motor symptoms (NMSS: 34.32 ± 21.41 vs. 30.11 ± 19.89, *p* = 0.041), with notable improvement in the gastrointestinal sub-item (3.79 ± 4.14 vs. 1.89 ± 2.54, *p* = 0.021). A reduction of IFN-γ levels was observed in both groups, but group A also showed a significant decrease in IL-6 and a slight increase in the anti-inflammatory cytokine TGF-β. **Conclusions.** Our data suggest that probiotics may modulate peripheral cytokines levels and improve clinical symptoms in PD patients. Probiotics may, therefore, represent a valuable adjunctive therapy to conventional anti-parkinsonian drugs.

## 1. Introduction

### 1.1. Background

Parkinson’s disease (PD) is a common neurodegenerative disorder characterized by bradykinesia, rest tremor, and rigidity [1]. The disease stems from the loss of dopaminergic neurons in the substantia nigra and the accumulation of a misfolded alpha-synuclein protein in surviving neurons [2]. Alpha-synuclein also accumulates in the gut, and according to Braak’s hypothesis, it may spread to the central nervous system via the vagus nerve [3], explaining why non-motor symptoms, like constipation, can precede the onset of classical motor symptoms for several years. Moreover, gut involvement in PD is supported by the evidence of different compositions of gut microbiota compared to healthy subjects; PD patients show increased levels of Enterobacteriaceae, which have been associated with more severe clinical presentations, including postural instability and gait disturbances [4]. The gut microbiota are increasingly recognized as key modulators of the central nervous system (CNS) by producing biogenic substances, such as GABA and serotonin, whose imbalances are linked to disease progression. Thus, restoring their levels could be a promising therapeutic target [5,6].

The pathophysiology of PD has not been fully understood, but it is clear that several factors should be taken into account, including age, gender, genetics, environment, and inflammation [7]. Neuroinflammation plays a critical role in PD pathophysiology, with inflammatory cells from both innate and adaptive immunity detected in both the periphery and CNS. Their interaction is reinforced by a disrupted brain-blood barrier (BBB) [8]. To date, it is unclear whether the dysregulation of the immune system may represent the primary cause of the neurodegenerative process or whether it represents an epiphenomenon.

It has been shown that aggregated alpha-synuclein can drive microglia toward a pro-inflammatory phenotype (M1 phenotype), thus creating a central pro-inflammatory environment [9]. Moreover, in an MPTP-induced murine model of PD, Brochard and colleagues demonstrated the presence of T lymphocytes in the substantia nigra [10]. At the same time, pro-inflammatory cells and cytokines may enter the CNS through the disrupted BBB, worsening the local hyper-inflammation and consequently leading to neuronal death [7]. Interestingly, several studies have demonstrated that patients with alpha-synucleinopaties (PD and multiple system atrophy-MSA) presented constitutionally reduced levels of anti-antibodies against alpha-synuclein, indicating how an impairment of immune functioning may predispose to these neurodegenerative diseases [11].

Dealing with PD, patients exhibit a peripheral pro-inflammatory immune phenotype, characterized by increased levels of Th1 and Th17 cells and a corresponding decrease of anti-inflammatory Th2 cells [12]. Furthermore, a dysregulation of the Treg compartment, involved in the counteract of the immune response, has been described as well. The dysregulation of the immune system has been demonstrated to contribute to the onset of both motor and non-motor symptoms, including REM-sleep behavior disorder, cognitive decline, and motor fluctuations [13,14,15].

The dysregulation of the immune system has also been studied in other atypical parkinsonism. Particularly, in their proteomic model, Dick et al. showed that the hyperactivation of immunological/inflammation-related pathways is more pronounced in prefrontal cortex tissue from patients with MSA compared to PD and progressive supranuclear palsy (PSP) [16]. Notwithstanding, a recent study by Alster P. and colleagues showed positive correlations between serum levels of pro-inflammatory cytokines like Interleukine (IL)1 and 6 with atrophy of specific brain regions in patients with PSP, thus indicating that neuroinflammation may be a driver of neurodegeneration also in this rare parkinsonism [17].

All these data together led to various clinical trials targeting the immune system in order to explore potential disease-modifying therapies [18].

### 1.2. Related Works

In this context, several preclinical studies have demonstrated that probiotics can interact with the immune system. Specifically, in murine models, probiotics have reduced peripheral pro-inflammatory cytokines such as interleukin (IL)1 beta and IL-6 [19,20]. In our previous study, we demonstrated that in peripheral mononuclear blood cells of PD patients, probiotics modulated the immune system by increasing anti-inflammatory cytokines, lowering pro-inflammatory cytokines, and reducing reactive oxygen species (ROS) [21]. This led to clinical trials showing that probiotics improve clinical motor (measured by the Unified Parkinson’s Disease Rating Scale) [22] and gastrointestinal symptoms [23,24,25]. Borzabadi and colleagues notably demonstrated that probiotics downregulated IL-1, IL-8, and tumor necrosis factor (TNF)-α gene expression in peripheral blood mononuclear cells (PBMC) of PD patients compared to healthy controls [26].

### 1.3. Aims of the Study

This study aims to evaluate the clinical and immunological effects of probiotic supplementation in a group of PD patients. Particularly, a group of PD patients were consecutively enrolled and blindly divided into two groups, one taking a mixture of probiotic strains while the other only maltodextrin. Patients were evaluated with specific clinical scales on both motor and non-motor symptoms at the beginning and end of the study, that is 12 weeks after enrollment. Furthermore, in these two time points, peripheral cytokines levels were assessed. The clinical evaluations will allow us to verify whether probiotics may have an impact on motor and non-motor symptoms of PD while the analysis of the peripheral cytokines will give an overview of the possible in vivo interaction of probiotics with these immune mediators and demonstrate the suitable action of shifting toward an anti-inflammatory profile.

## 2. Materials and Methods

### 2.1. Patients

This study was approved by the local Ethics Committee (CE 216/21). Patients were included in the study after providing informed consent. A total of 40 PD patients, regularly followed at the Movement Disorders Centre in Novara, were enrolled and randomly assigned to one of two groups in a blinded manner. Group A received a product containing a mix of probiotics: Bifidobacterium animalis subsp. lactis BS01 (≥1 × 109 CFU/AFU), Bifidobacterium longum 03 (≥1 × 109 CFU/AFU), Bifidobacterium adolescentis BA02 (≥1 × 109 CFU/AFU), Fructo-oligosaccharides FOS (2500 mg) and maltodextrin (q.s). Group B received only maltrodextrin (q.s).

**Figure 1:**
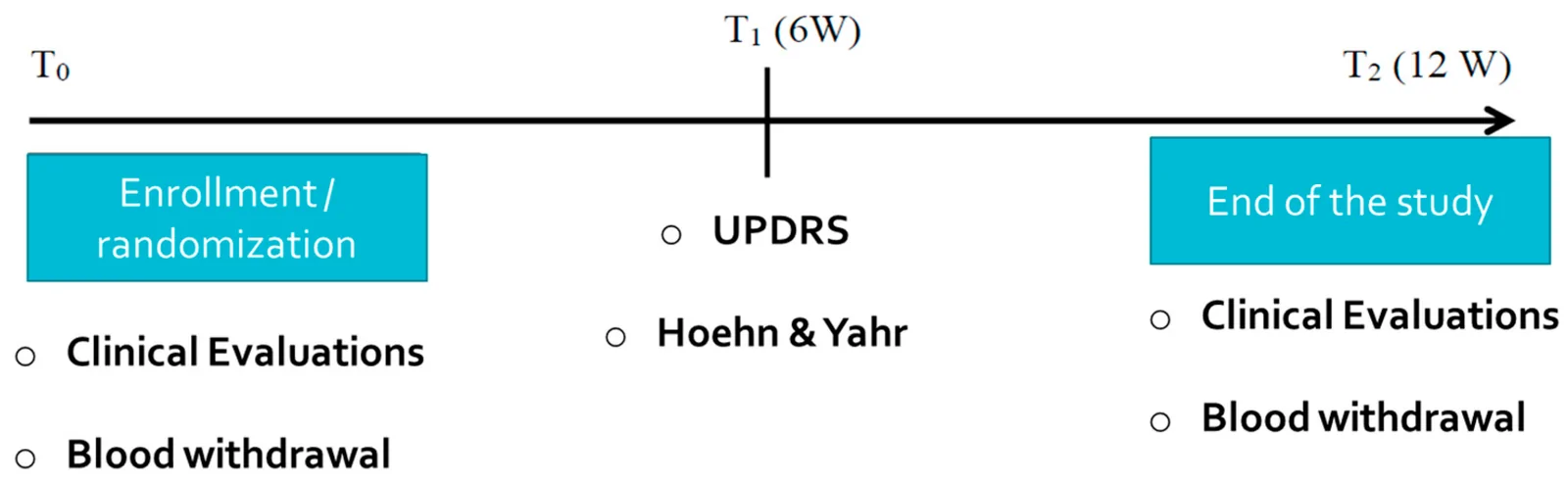
Schematic representation of the study protocol.

The study protocol included three time points, as shown in Figure 1.

At baseline (T0) and at the end of the study (T2), blood samples were collected to assess circulating cytokine levels. At these two time points, patients were evaluated by a movement disorders specialist using specific scales for motor and non-motor symptoms. The assessment included the Unified Parkinson’s Disease Rating Scale (UPDRS), Hoehn and Yahr stage (H&Y), Zung self-rating Anxiety Scale, Beck Depression Inventory Scale (BDI-II), Composite Autonomic Symptoms Scale-31 (COMPASS 31), Montreal Cognitive Assessment (MOCA) PAC-QOL, Non-Motor Symptoms Scale (NMSS), Wexner Scale, Constipation Assessment Scale (CAS), and Bristol Stool Form Chart. At T1 (6 weeks after the study began) patients were evaluated for PD symptoms using UPDRS and H&Y stage.

### 2.2. Choice of Probiotic Strains

The selection of probiotic strains (BA02, BL03, and BS01) was based on their capacity to produce GABA, supported by the bioinformatic screening of GABA-related genes. Beyond GABA production, these strains have genes related to critical probiotic traits, including serotonin modulation, degradation of anti-nutritional factors (ANFs), adhesion to intestinal surfaces, resistance to gastrointestinal tract conditions (GIT), and the presence of the *abfA* gene cluster, associated with improved gastrointestinal motility. In previous work [21], we demonstrated that BS01 significantly counteracted pro-inflammatory cytokines production in PBMC of PD patients.

### 2.3. Cytokines Levels Evaluation

Blood samples were centrifugated at 1400× *g* for 10 min at room temperature. Plasma aliquots were stored at −80 °C and thawed at 4 °C immediately prior to analysis. Before analysis, the samples were brought to room temperature. A fixed aliquot of 50 μL of the sample was used to evaluate circulating cytokine concentrations, including interferon (IFN)-γ, IL-6, and TNF-α, via a multiplex magnetic bead-based assay (Bio-Plex Pro Human Cytokine Assay, Bio-Rad Laboratories, Inc., Hercules, CA, USA), according to the manufacturer’s instructions. Analyses were performed on a Bio-Plex 200 System (Bio-Rad) using Bio-Plex Manager software version 6.2.0.175. TGF-β1 was assessed in a separate panel using the same multiplex system.

### 2.4. Statistical Analysis

Statistical analyses were performed using parametric (*t*-test for independent and paired samples) or non-parametric (Mann–Whitney or Wilcoxon) tests according to the normal distribution of the data evaluated with the Shapiro–Wilk test. Categorical variables are reported as counts (percentages), while continuous variables are expressed as mean (standard deviation, SD) or median (interquartile range, IQR) based on data distribution. All tests were two-tailed, with a significance threshold of *p* < 0.05. Statistical analyses were performed using SPSS version 25 (IBM Corporation, Armonk, NY, USA).

## 3. Results

### 3.1. Study Population

A total of 40 PD patients (31 males) were initially enrolled, though 2 patients (one in each group) dropped out: 1 due to SARS-CoV-2 infection, and the other refused to continue the study. For the first patient who dropped out, only clinical data were collected. The mean age at enrollment was 68.4 (±7.3) years, with a mean age at disease onset at 64.5 (±7.0) years. The Levodopa equivalent daily dose (LEDD) was calculated for each patient according to Tomlinson and Cilia et al. [27,28]. Demographic and baseline characteristics of the two groups were comparable (Table 1 and Table 2). Information about comorbidities and ongoing pharmacological therapies was collected for all patients as well, and no differences were detected between the two groups (See Supplementary Tables S1 and S2). As shown in Table 1, our patients were mostly men: this can be partially due to the general increased prevalence of the disease among men but also to the consecutive enrollment of patients at the Center. Nevertheless, despite this aspect, the two groups were comparable regarding gender.

### 3.2. Motor and Non-Motor Symptoms

Patients in Group A showed a significant improvement in motor symptoms, indicated by a decrease in UPDRS score (13.89 ± 4.08 vs. 12.74 ± 4.57 *p* = 0.028) (Table 3). The H&Y stage remained unchanged in both groups. The impact on motor symptoms may also be indirect: by promoting intestinal transit, probiotics may permit a better absorption of the therapy, which, in turn, exerts a more pronounced clinical impact.

No significant effects were detected in the Zung and BDI-II scales. Particularly considering the BDI-II scale, Group A patients presented a slight mean reduction while the total score remained stable in Group B. This improvement may also be related to the positive effects on motor symptoms and the gastrointestinal discomfort commonly afflicting PD patients.

Concerning non-motor symptoms, the total NMSS score significantly decreased in Group A (34.32 ± 21.41 vs. 30.11 ± 19.89 *p* = 0.041). Within the NMSS subitems, Group A patients demonstrated a notable improvement in gastrointestinal symptoms (3.79 ± 4.14 vs. 1.89 ± 2.54, *p* = 0.021) (Table 4). The positive impact of probiotics on gastrointestinal symptoms was also detected, even if not statistically significant, as a positive trend in the gastrointestinal discomfort in the Compass 31 scale subitems (7.68 ± 4.41 vs. 6.21 ± 3.94; Supplementary Table S3) and a reduction in the social discomfort subitem in the PaC-QoL scale (6.11 ± 6.03 vs. 4.84 ± 5.29 *p* = 0.074); Supplementary Table S4). Notably, these data support the suitable positive effects of probiotics on non-motor symptoms in PD. As for the motor symptoms, the effects of probiotics may be indirect: this positive effect may indeed be the indirect consequence of better intestinal functioning with a more pronounced effect of the pharmacological therapy.

When analyzing the cognitive domain, patients taking probiotics showed a slight improvement in the MOCA score, while Group B exhibited a mild decline (27.47 ± 1.81 vs. 28.05 ± 1.68 *p* = 0.078; 26.30 ± 2.77 vs. 25.70 ± 2.98 *p* = 0.065, respectively). Additionally, a positive trend in the cognitive subitem of the NMSS was observed (4.11 ± 6.76 vs. 3.11 ± 6.19 *p* = 0.058) in Group A.

Dealing with constipation, a main issue for PD patients, no significant variations were detected before and after probiotic treatment. However, a significant reduction of the CAS score has been detected in Group B, which, on one side, may be a placebo effect, and on the other, may be the prebiotic effect of maltodextrin. Notwithstanding, Group A showed a trend in reduction of the Wexner total score, indicating a slight improvement in constipation.

### 3.3. Circulating Cytokine Levels

Baseline levels of IFN-γ, IL-6, TNFα, and Transforming Growth Factor β (TGF-β) did not differ in the two groups. Both groups showed a statistically significant reduction of IFN-γ, while a significant decrease in IL-6 levels was observed only in Group A. Moreover, although not statistically significant, only patients in the probiotics group showed a slight increase in the anti-inflammatory cytokine TGF-β, whose levels remained stable in Group B (Table 5). All these data together show a positive effect of the probiotics on the peripheral cytokines, promoting a significant reduction of the pro-inflammatory ones and a slight increase of the anti-inflammatory ones.

## 4. Discussion 

The results of this study provide evidence that probiotic supplementation can improve both motor and non-motor symptoms in patients with PD, particularly in areas related to gastrointestinal health, mood, and cognition. Furthermore, this study highlights the potential anti-inflammatory effect of probiotic therapy, as reflected by changes in circulating cytokine levels.

The observed improvements in motor symptoms in the probiotic group are noteworthy. While PD motor symptoms are primarily linked to dopamine deficiency due to the loss of dopaminergic neurons, emerging evidence suggests that systemic inflammation and gastrointestinal dysfunction can exacerbate motor impairment [29]. The reduction in UPDRS motor scores among patients receiving probiotics may suggest an indirect, beneficial effect of gut microbiota modulation on motor function. Previous research supports this hypothesis, with studies demonstrating that gut microbiota alterations can influence motor symptomatology in PD by impacting systemic inflammation and neurotransmitter synthesis [30,31]. Moreover, dysregulation of GABAergic signaling may be implicated in both motor and non-motor symptoms of PD, making it a relevant therapeutic target [32]. For instance, the GABAA agonist Zolpidem has demonstrated efficacy in reducing dyskinesia [33], while the GABAA antagonist flumazenil has improved postural stability by modulating brainstem inhibitory tone [34]. Accordingly, the administration of probiotics that are able to enhance GABA production may have a relevant clinical impact.

Non-motor symptoms in PD, including gastrointestinal, cognitive, and mood-related symptoms, significantly affect quality of life and often precede the onset of motor symptoms [35]. In this study, patients receiving probiotics showed a marked reduction in gastrointestinal discomfort, as reflected in the NMSS gastrointestinal subscore. This finding aligns with previous studies that have reported improvements in gastrointestinal symptoms, such as constipation, in PD patients following probiotic supplementation [23,36,37]. It has been hypothesized that probiotics may enhance gastrointestinal motility by modulating the enteric nervous system and through the production of short-chain fatty acids (SCFAs) that stimulate gut function [38].

A modest improvement in cognitive function, as measured by the MOCA score, was observed in the probiotic group, along with a positive trend in the NMSS cognitive subitem. These findings are consistent with preclinical evidence showing that probiotics can modulate neurotransmitters such as GABA, serotonin, and dopamine, which are involved in cognitive processes [39]. Particularly, it has been shown that GABA may exert positive cognitive benefits: in experimental models, administration of valproate, which enhanced GABA release, determined a significative improvement of cognitive function [40].

Additionally, the gut-brain axis is increasingly recognized as a potential target for interventions aiming to slow cognitive decline in neurodegenerative diseases. The presence of the *abfA* gene in the selected probiotic strains, which is linked to enhanced gut motility and nutrient absorption [41], might play a role in this observed improvement in cognition by ensuring optimal nutrient availability for brain function.

Mood-related improvements, specifically a trend toward reduced anxiety and depression, were also noted in the probiotic group. Although the changes were not statistically significant, these findings are in line with the growing literature on the “psychobiotic” potential of probiotics—strains capable of positively affecting mood and mental health [42]. Probiotics have been shown to influence brain function via the production of neurotransmitters and by modulating the hypothalamic-pituitary-adrenal (HPA) axis, which regulates stress responses [43].

One of the most compelling aspects of this study is the reduction in pro-inflammatory cytokines, specifically IFN-γ and IL-6, in the probiotic group. These cytokines are known to be elevated in PD patients and are thought to contribute to the progression of neuroinflammation and neurodegeneration. The ability of probiotics to modulate immune responses by reducing pro-inflammatory and potentially increasing anti-inflammatory cytokines supports their role as an adjunctive therapy in PD. In previous studies, the BS01 strain has been shown to downregulate pro-inflammatory pathways in PBMCs of PD patients, which may explain the reduction in IFN-γ and IL-6 observed here [21]. Furthermore, Borzobadi et al. reported a decrease in IL-1, IL-8, and TNF-α gene expression alongside an increase in TGF-β and PPAR-γ gene expression after 12 weeks of probiotic intake [26]. Similar effects have been observed in other conditions, such as major depressive disorder, where a four-week course of probiotics significantly reduced IL-6 gene expression in patients with depression [44].

In the control group (Group B) receiving maltodextrin, there was a more pronounced reduction in CAS scores, possibly due to biases in self-reported questionnaires. However, maltodextrin, as a prebiotic, has also been shown to positively influence gastrointestinal homeostasis, notably by reducing constipation [45]. Group B participants also exhibited a significant reduction in IL-6, consistent with findings in type 2 diabetes patients on maltodextrin supplementation [46].

However, despite the promising results presented, several limitations of our work should be considered. Though similar to other published trials, the sample size was relatively small, thus limiting the statistical power to detect all potential effects of probiotic therapy in PD. Moreover, the duration of the intervention was relatively short, but it was similar to other previously published studies in which positive results about the probiotics effect have been described. However, it is unclear whether the benefits observed could be sustained over a longer period. Therefore, longer studies are needed.

Future studies should explore the long-term effects of probiotics on PD symptoms and their underlying mechanisms, including the specific roles of different microbial strains in neuroinflammation and neurotransmitter modulation.

## 5. Conclusions

In conclusion, our findings suggest that probiotics could be a promising adjunctive therapy for managing both motor and non-motor symptoms in PD. By improving gastrointestinal function, modulating immune responses, and potentially influencing neurotransmitter levels, probiotics may address several pathophysiological mechanisms underlying PD. Further large-scale studies are needed to confirm these effects and to explore the optimal strains, dosages, and treatment durations for probiotic therapy in PD.

## Figures and Tables

**Table 1:** Demographic characteristics of the two groups.

	Group A	Group B	*p*-Value Group A vs. Group B
Age (years, mean ± SD)	67.32 ± 7.03	69.40 ± 7.51	0.243
Age at PD onset (years, mean ± SD)	63.53 ± 6.92	65.35 ± 7.22	0.338
Disease duration (years, mean ± SD)	3.79 ± 1.18	4.05 ± 0.94	0.546
LEDD (mg/die, mean ±SD)	647.68 ± 239.36	426.60 ± 160.82	0.800
Gender (males, *n*; %)	14 (74)	17 (85)	0.451
Anti-parkinsonian therapy (n, %) Levodopa Dopamine-agonists MAO-B inhibitors COMT inhibitors Anti-cholinergic	17 (89.5) 10 (52.6) 15 (78.9) 0 0	16 (80) 8 (40) 14 (70) 1 (5) 1 (5)	0.661 0.527 0.716 1 1

**Table 2:** Clinical characteristics of the study population.

	Group A	Group B	*p*-Value Group A vs. Group B
UPDRS	13.89 ± 4.08	15.05 ± 4.41	0.331
H&Y	1.95 ± 0.40	2.05 ± 0.43	0.306
Zung	32.05 ± 7.83	34.05 ± 7.63	0.367
BDI—II	8.00 ± 4.26	7.75 ± 7.05	0.296
Compass 31 Total Orthostatic intolerance Vasomotor Secretomotor Gastrointestinal Bladder Pupillomotor	17.26 ± 9.03 1.68 ± 2.52 0.42 ± 1.02 1.74 ± 1.52 7.68 ± 4.41 1.42 ± 1.89 4.32 ± 3.27	14.15 ± 8.00 1.80 ± 2.44 0.20 ± 0.89 1.00 ± 1.38 5.80 ± 3.21 1.45 ± 1.39 3.90 ± 3.37	0.267 0.860 0.311 0.082 0.119 0.578 0.617
MOCA	27.47 ± 1.81	26.30 ± 2.77	0.222
PAC-QoL Total Physical discomfort Social discomfort Worries Satisfaction	43.89 ± 18.85 7.58 ± 3.13 6.11 ± 6.03 21.68 ± 10.37 8.53 ± 3.31	38.15 ± 13.87 6.20 ± 2.17 4.75 ± 4.85 19.55 ± 7.45 7.65 ± 3.67	0.383 0.191 0.472 0.735 0.329
NMSS Total Cardiovascular Sleep/fatigue Mood/cognition Perceptual problems/hallucinations Attention/memory Gastrointestinal tract Urinary Sexual function Miscellaneous	34.32 ± 21.41 1.05 ± 1.61 7.26 ± 6.52 4.11 ± 6.76 0.37 ± 0.83 3.58 ± 2.55 3.79 ± 4.14 8.63 ± 7.30 1.21 ± 1.96 4.37 ± 4.41	42.65 ± 36.43 1.25 ± 2.17 7.95 ± 7.28 8.15 ± 14.66 0.75 ± 1.16 5.15 ± 6.47 4.05 ± 4.32 9.35 ± 9.72 1.40 ± 2.09 4.60 ± 4.55	0.746 0.889 0.955 0.584 0.287 0.955 0.765 0.855 0.865 0.799
CAS	4.05 ± 3.64	2.00 ± 2.10	0.066
Wexner	7.79 ± 5.43	7.20 ± 4.84	0.757

**Table 3:** Comparison of the main results between the two groups at the beginning and the end of the study.

	Group A			Group B		
	T0	T2	Z; *p*	T0	T2	Z; *p*
UPDRS	13.89 ± 4.08	12.74 ± 4.57	−2.201; 0.028	15.05 ± 4.41	14.35 ± 4.25	−1.728; 0.084
H&Y	1.95 ± 0.40	1.95 ± 0.40	0; 1	2.05 ± 0.43	2.05 ± 0.43	0; 1
Zung	32.05 ± 7.83	32.42 ± 6.20	−0.315; 0.753	34.05 ± 7.63	35.00 ± 7.36	−0.882; 0.378
BDI-II	8.00 ± 4.26	6.79 ± 4.92	−1.523; 0.128	7.75 ± 7.05	7.95 ± 7.74	−0.141; 0.888
Compass-31 total	17.26 ± 9.03	15.23 ± 8.18	−1.114; 0.265	14.15 ± 8.00	14.35 ± 9.04	−0.308; 0.758
Pac-QoL total	43.89 ± 18.85	41.11 ± 19.17	−0.935; 0.351	38.15 ± 13.87	34.90 ± 11.82	−1.492; 0.136
MOCA	27.47 ± 1.81	28.05 ± 1.68	−1.761; 0.078	26.30 ± 2.77	25.70 ± 2.98	−1.847; 0.065
NMSS total	34.32 ± 21.41	30.11 ± 19.89	−2.043; 0.041	42.65 ± 36.43	39.60 ± 29.03	−0.628 ± 0.530
CAS	4.05 ± 3.64	2.32 ± 2.06	−1.692; 0.091	2.00 ± 2.10	1.05 ± 1.28	−2.612; 0.009
Wexner	7.79 ± 5.43	6.37 ± 4.23	−1.380; 0.168	7.20 ± 4.84	7.15 ± 5.58	−0.229; 0.819

**Table 4:** Non-motor symptoms scale (total and subitems) score of the two groups at the beginning and end of the study.

	Group A			Group B		
	T0	T2	Z; *p*	T0	T2	Z; *p*
NMSS total	34.32 ± 21.41	30.11 ± 19.89	−2.043; 0.041	42.65 ± 36.43	39.60 ± 29.03	−0.628; 0.530
Cardiovascular	1.05 ± 1.61	1.21 ± 2.18	−0.744; 0.457	1.25 ± 2.17	0.80 ± 1.15	−1.051; 0.293
Sleep/fatigue	7.26 ± 6.52	7.26 ± 6.35	−0.306; 0.760	7.95 ± 7.28	6.70 ± 6.67	−1.298; 0.194
Cognition	4.11 ± 6.76	3.11 ± 6.19	−1.897; 0.058	8.15 ± 14.66	7.75 ± 11.11	−0.438; 0.662
Hallucinations	0.37 ± 0.83	0.32 ± 0.95	0; 1	0.75 ± 1.16	1.05 ± 3.19	−0.272; 0.785
Attention	3.58 ± 2.55	3.26 ± 2.42	−0.813; 0.416	5.15 ± 6.47	5.40 ± 5.44	−0.519; 0.874
Gastrointestinal	3.79 ± 4.14	1.89 ± 2.54	−2.299; 0.021	4.05 ± 4.32	3.70 ± 4.23	−0.975; 0.329
Urinary	8.63 ± 7.30	7.84 ± 6.73	−0.847; 0.397	9.35 ± 9.72	8.80 ± 8.42	−0.313; 0.755
Sexual	1.21 ± 1.96	0.89 ± 1.94	−1.604; 0.109	1.40 ± 2.09	1.15 ± 1.76	−0.120; 0.905
Miscellaneous	4.37 ± 4.41	4.89 ± 5.98	−0.631; 0.528	4.60 ± 4.55	4.25 ± 4.12	−0.580; 0.562

**Table 5:** Levels of circulating cytokines at the beginning and end of the study.

	Group A			Group B		
	T0	T2	*p*	T0	T2	*p*
IFN γ	4.26 ± 2.52	1.78 ± 2.46	<0.001	4.68 ± 2.49	2.14 ± 1.91	<0.001
IL6	1.14 ± 0.70	0.79 ± 0.60	0.007	0.92 ± 0.34	0.72 ± 0.57	0.06
TNF α	3.73 ± 1.62	4.38 ± 2.71	0.11	3.96 ± 1.72	4.08 ± 1.47	0.44
TGFβ	37.48 ± 11.1	42.35 ± 16.2	0.18	36.31 ± 11.2	35.3 ± 13.85	0.85

## Data Availability

The data presented in this study are available on request from the corresponding author.

## Supplementary Materials

The following supporting information can be downloaded at: https://www.mdpi.com/article/10.3390/brainsci14111147/s1; Table S1: Comorbidities of the patients included in the study; Table S2: Other pharmacological therapies ongoing during the study apart from anti-parkinsonian drugs; Table S3: Compass-31 scale (total and subitem) score of the two groups at the beginning and end of the study, Table S4: PaC-QoL scale (total and subitem) score of the two groups at the beginning and end of the study.
