# Correction: Magistrelli et al. The Impact of Probiotics on Clinical Symptoms and Peripheral Cytokines Levels in Parkinson’s Disease: Preliminary In Vivo Data. *Brain Sci.* 2024, *14*, 1147

**DOI:** 10.3390/brainsci16070715

**Published:** 2026-07-03

**Authors:** Luca Magistrelli, Elena Contaldi, Annalisa Visciglia, Giovanni Deusebio, Marco Pane, Angela Amoruso

**Affiliations:** 1 Parkinson Institute Milan, ASST G.Pini-CTO, Via Bignami 1, 20126 Milan, Italy; contaldie@yahoo.it; 2 Probiotical Research S.r.l., Via Mattei 3, 28100 Novara, Italy; a.visciglia@probiotical.com (A.V.); g.deusebio@probiotical.com (G.D.); m.pane@probiotical.com (M.P.); a.amoruso@probiotical.com (A.A.)

Following the publication of this article [1], the authors would like to update the Institutional Review Board Statement and Conflicts of Interest to clarify details.

**Institutional Review Board Statement:** The study was conducted in accordance with the Declaration of Helsinki and approved by the Ethics Committee of Novara (protocol code CE 216/21, approved on 7 October 2021). Dr. Luca Magistrelli is indicated as the PI of the study. The study was subsequently registered on clinicaltrial.gov in November 2021 by Prof. Franca Marino (Centre for Research in Medical Pharmacology), also including a flow cytometry analysis. The data presented herein constitute a preliminary sub-analysis and were all collected and entirely analyzed by the UPO-CAAD team in Novara under the supervision of the PI of the study, as stated in the approval of the Ethical Committee of Novara.

**Conflicts of Interest:** Probiotical Research is a legal entity, part of the Probiotical group and wholly owned by Probiotical S.p.A., whose core activity is manufacturing. Within this corporate structure, Probiotical Research operates as a research department that is responsible for investigating probiotic solutions that may subsequently be produced by Probiotical S.p.A. Probiotical Research played a decisive scientific role only in the formulation of the supply for the clinical study. Nevertheless, Probiotical S.p.A. and Probiotical Research reaffirm that neither entity has provided any funding for the clinical study other than supplying the probiotic product at no cost. Probiotical S.p.A. hereby states that it has provided no monetary funding or financial support to the Principal Investigator (“PI”) of the referenced clinical study. Probiotical S.p.A.’s involvement was strictly limited to the free-of-charge and experimental supply of both the active product and the placebo. Probiotical Research had no role whatsoever in the study design, data collection, data analysis, statistical processing, or interpretation. All the scientific analyses were performed by the academic and clinical team at the UPO-CAAD in Novara. Probiotical Research’s contribution was strictly logistical (physical handling and transport of anonymized specimens and coverage of minor logistics-related costs, such as packaging, courier shipment, and routine consumables) and did not involve access to identifiable data or source datasets. As such, no human-subject research was conducted at Probiotical and no patient-level data were processed there.

The authors state that the scientific conclusions are unaffected. These corrections were approved by the Academic Editor. The original publication has also been updated.
