# Supplementary material for: The Impact of Probiotics on Clinical Symptoms and Peripheral Cytokines Levels in Parkinson’s Disease: Preliminary In Vivo Data
Source: Brain Sci. 2024 Nov 15;14(11):1147. doi: 10.3390/brainsci14111147 (PMC11592242; doi:10.3390/brainsci14111147)
Supplement: Supplementary file 1 [file brainsci-14-01147-s001.zip › brainsci-3316120-supplementary.pdf]

Supplementary Table S1. Comorbidities of the patients included in the study

**Comorbidities, number of patients (%)**	**Group A (N=20)**	**Group B (N = 20)**	**P-value Group A *vs* Group B**
Hypertension	8 (40%)	10 (50%)	0.751
Gastroesophageal reflux	1 (5%)	5 (25%)	0.182
Dyslipidemia	2 (10%)	7 (35%)	0.127
Diabetes	2 (10%)	4 (20%)	0.052
Hearth disease	3 (15%)	4 (20%)	1.0
Depression	4 (20%)	5 (25%)	1.0
Benign prostatic hypertrophy	4 (20%)	2 (10%)	0.407
Hypothyroidism	3 (15%)	0	0.106

Supplementary Table S2. Other pharmacological therapies ongoing during the study apart from anti-parkinsonian drugs

**Treatment, number of patients (%)**	**Group A (N=20)**	**Group B (N=20)**	**P-value Group A *vs* Group B**
PPI	1 (5%)	4 (20%)	0.342
Anti-hypertensive	11 (55%)	7 (35%)	0.205
Anti-platelet	5 (25%)	4 (20%)	0.716
Hypoglycemic	2 (10%)	2 (10%)	1.0
Statins	1 (5%)	4 (20%)	0.342
Anti benign prostatic hypertrophy	4 (20%)	1 (5%)	0.182
Anti-arrhythmic	1 (5%)	1 (5%)	1.0
Anti-coagulants	1 (5%)	0	0.487
Anti-depressants	3 (15%)	2 (10%)	0.661
Levothyroxine	3 (15%)	0	0.106

Supplementary Table S3 Compass-31 scale (total and subitem) score of the two groups at the beginning and end of the study

	**Group A**			**Group B**		
	T0	T2	Z; p	T0	T2	Z; p
Total score	17.26±9.03	15.32±8.18	-1.114;0.265	14.15±8.00	14.35±9.04	-0.308;0.758
Orthostatic intolerance	1.68±2.52	1.37±2.14	-0.954;0.340	1.80±2.44	1.85±2.48	-0.060;0.952
Vasomotor function	0.42±1.02	0.95±1.47	-1.642;0.180	0.20±0.89	0.45±1.10	-0.447;0.655
Secretomotor function	1.74±1.52	1.11±1.63	-1.633;0.102	1.00±1.38	0.95±1.61	-0.213;0.832
Gastrointestinal function	7.68±4.41	6.21±3.94	-1.483;0.138	5.80±3.21	5.60±3.45	-0.459;0.646
Bladder	1.42±1.89	1.37±1.21	-0.144;0.886	1.45±1.39	1.60±1.57	-0.905;0.366
Pupillomotor	4.32±3.27	4.32±3.06	-0.179;0.858	3.90±3.37	3.90±3.40	-0.039;0.969

Supplementary Table S4 PaC-QoL scale (total and subitem) score of the two groups at the beginning and end of the study

	Group A			Group B		
	T0	T2	Z; p	T0	T2	Z; p
Total score	43.89±18.85	41.11±19.17	-0.932;0.351	38.51±13.87	34.90±11.82	-1.492;0.136
Physical discomfort	7.58±3.13	7.11±4.04	-1.460;0.144	6.20±2.17	6.00±2.25	-0.396;0.692
Psychosocial discomfort	6.11±6.03	4.84±5.29	-1.785;0.074	4.75±4.85	4.40±3.41	-0.141;0.888
Worries and concerns	21.68±10.37	19.79±9.94	-1.369;0.171	19.55±7.45	16.70±6.26	-1.994;0.046
Satisfaction	8.53±3.31	9.37±3.74	-1.264;0.206	7.65±3.67	7.80±2.82	0.000;1.00
